# Editorial: The increasing relevance of traditional medicine systems for the primary health care sector and general practice: global research perspectives

**DOI:** 10.3389/fmed.2024.1533361

**Published:** 2025-01-07

**Authors:** Christian S. Kessler, Pathirage Kamal Perera, Rammanohar Puthiyedath, Anand Dhruva

**Affiliations:** ^1^Department of Internal Medicine and Nature-Based Therapies, Immanuel Hospital Berlin, Berlin, Germany; ^2^Charité Competence Center for Traditional and Integrative Medicine (CCCTIM), Charité – Universitätsmedizin Berlin, Corporate Member of Freie Universität Berlin, Humboldt-Universität zu Berlin and Berlin Institute of Health, Berlin, Germany; ^3^Faculty of Indigenous Medicine, University of Colombo, Colombo, Sri Lanka; ^4^Amrita School of Ayurveda, Amrita Vishwa Vidyapeetham, Amritapuri, Kerala, India; ^5^Department of Medicine, Division of Hematology and Oncology, University of California, San Francisco, San Francisco, CA, United States; ^6^Osher Center for Integrative Health, University of California, San Francisco, San Francisco, CA, United States

**Keywords:** traditional medicine, complementary medicine, integrative medicine, primary healthcare, family medicine

## Introduction

In the context of global health care challenges, traditional, complementary and integrative medicine (TCIM) systems have gained increasing attention due to their potential to improve primary health care and beyond (1). This editorial addresses the importance of TCIM in addressing unmet health care needs, particularly in the primary care setting. As traditional medicine gains greater recognition worldwide, initiatives such as the recent WHO Summit on Traditional Medicine—culminating in the Gujarat Declaration (2)—highlight the value of integrating TCIM approaches on a global scale. These summits advocate for an evidence-informed and culturally sensitive incorporation of TCIM to improve health outcomes and at the same time recognize the long-standing role of traditional practices in health care and for wellbeing across diverse populations (3).

The body of research presented in this Research Topic highlights the role of TCIM in addressing diverse health issues. Studies range from analyzing the efficacy of traditional herbal medicines to exploring TCIM approaches that support holistic health, providing global perspectives that shape our understanding of the potential of TCIM in modern healthcare settings.

## Overview of featured research articles

1. *Exploring the association between phytopharmaceutical use and antibiotic prescriptions in upper respiratory infections: results from a German cohort study evaluating the impact of naturopathy qualifications of general practitioners using routine data* (Wetzel et al.) investigates the influence of phytopharmaceutical prescriptions on antibiotic use. This German study found that general practitioners with TCIM qualifications prescribed fewer antibiotics, illustrating the potential of TCIM to support antibiotic stewardship in primary care settings.
2. *Efficacy and safety of Lianhua Qingwen granule in the treatment of non-influenza viral pneumonia: a randomized, double-blind, placebo-controlled, multicenter clinical study* (Ma et al.) presents results of a clinical trial that suggests efficacy and safety of Lianhua Qingwen in relieving symptoms of viral pneumonia. This study highlights the potential role of traditional Chinese medicine (TCM) in managing infectious respiratory conditions, an area with heightened relevance post COVID-19.
3. *Associations of traditional Chinese medicine body constitution and all-cause mortality in patients with type 2 diabetes mellitus: a prospective cohort study of a Taiwanese medical center* (Lee et al.) examines associations between TCM body constitution types and mortality in diabetic patients, suggesting that Yin deficiency correlates with higher mortality risk. This work suggests that TCM could inform tailored diabetes management strategies.
4. *Efficacy and safety of the Ayurvedic herbal preparation Maharishi Amrit Kalash: a systematic review of randomized controlled trials* (Koch et al.) systematically reviews MAK, an Ayurvedic supplement, in supporting chemotherapy side effects and cognitive function, encouraging further research into Ayurveda's potential therapeutic roles within integrative oncology.
5. *Can acupuncture increase microcirculation in peripheral artery disease and diabetic foot syndrome? – a pilot study* (Valentini et al.) shows promising results of acupuncture in enhancing microcirculation for diabetic patients, underscoring TCIM's potential role in managing common chronic complications of diabetes mellitus and improving quality of life.
6. *Use and acceptance of traditional, complementary and integrative medicine in Germany—an online representative cross-sectional study* (Jeitler et al.) provides an overview of TCIM's widespread use in Germany, with a significant percentage of the population endorsing it as relevant to their health. These findings stress the need for health policies that recognize TCIM's role in meeting patient needs.
7. *Development of the Korean Medicine Core Outcome Set for Facial Palsy: herbal medicine treatment of patients with facial palsy in primary clinics* (Kim, Kim et al.) contributes a core outcome set for herbal treatment of facial palsy, aligning TCIM evaluation methodologies with mainstream medical standards.
8. *A review of the WHO strategy on traditional, complementary, and integrative medicine from the perspective of academic consortia for integrative medicine and health* (Hoenders et al.) looks at the WHO's TCIM strategies from the lens of academic consortias, arguing for an enhanced integrative approach that builds on both biomedical and TCIM practices for improved healthcare outcomes.
9. *Wellness or medicine? Use and perception of Ayurveda in Germany: data from an online-representative cross-sectional study* (Schiele et al.) examines the use and perception of Ayurveda in Germany. The survey results suggest a growing interest in its medical application, with acceptance being limited by cultural differences and a lack of scientific evidence.
10. *Exploring the gap: attitudes, knowledge, and training needs in complementary and integrative medicine among healthcare professionals at German university hospitals* (Hesmert et al.) underscores the importance of training for healthcare professionals to effectively integrate TCIM into patient care, addressing a gap that is essential for integrative patient management.
11. *Protocol for a scoping review of traditional medicine research methods, methodologies, frameworks and strategies* (Ijaz et al.) lays out a scoping review protocol to explore methodologies suited to TCIM research, advocating for an approach to bridge both indigenous and biomedical knowledge systems.
12. *Comprehensive review of Korean Medicine registries 2015–2023* (Kim, Choi et al.) highlights the role of registries in capturing real-world TCIM data, suggesting that registries could inform policy and practice on TCIM's effectiveness and safety in chronic disease management.
13. *Effect of the health and wellness Kneipp concept on health promotion and reduction of sick days for kindergarten children: a cluster randomized controlled trial protocol* (Gerganova et al.) outlines a study protocol evaluating the Kneipp health concept in children, a TCIM intervention with potential to improve child health through lifestyle-based therapies.
14. *Integrative nursing interventions: knowledge, attitudes and practice in home nursing services in Germany—a quantitative and qualitative online survey* (Stolz et al.) explores how integrative nursing interventions, like aromatherapy and herbal teas, are applied in home care settings, reinforcing the importance of TCIM in chronic care.
15. *Effectiveness and safety of acupuncture modalities for overweight and obesity treatment: a systematic review and network meta-analysis of RCTs* (Kim Y. et al.) presents a systematic review and meta-analysis on acupuncture's efficacy in managing obesity, pointing at its role as a complementary approach to weight management in TCIM.

## The global relevance of TCIM in healthcare

The works presented highlight the multiple potential applications and benefits of TCIM and underscore its value as a patient-centered approach (4). The WHO summit on TCIM has accelerated efforts to systematically integrate evidence-informed TCIM into global healthcare systems, recognizing its cultural relevance and its cost-effective contribution to health outcomes, particularly in underserved areas. In Germany, for example, acceptance of TCIM is high, indicating a cultural need and readiness for integrative health policies that include both biomedicine and TCIM practices (Jeitler et al.). Continued research, education, and policy integration of TCIM are critical to promoting a health paradigm that values both traditional and scientific knowledge systems (Hoenders et al.). These efforts support the vision of the WHO Gujarat Declaration on TCIM, which advocates for an integrative approach to health care that respects traditional wisdom while at the same time advancing modern health practices (2).

## Conclusion

As TCIM obviously gains momentum globally, its integration into primary health care could address critical gaps in healthcare delivery, particularly in the areas of chronic disease management, preventive care and health promotion. The studies presented here support the growing role of TCIM in global health, and future research will be critical to its further development and scientific validation in healthcare systems. This Research Topic is a testament to the potential of TCIM to improve health care accessibility, promote sustainable practices, and enrich the diversity of health care knowledge. The papers in this Research Topic not only highlight the therapeutic potential of TCIM, but also emphasize the need for robust scientific methods to validate and integrate these practices into modern healthcare, in line with the sustainable development goals of the WHO (5).
